# Perinatal findings in a patient with a novel large chromosome 19p deletion

**DOI:** 10.1002/ccr3.1615

**Published:** 2018-06-21

**Authors:** Marko Culjat, Jennifer Razak, Reem Saadeh‐Haddad, Rita Driggers, Karen Kamholz, Julia Timofeev

**Affiliations:** ^1^ MedStar Georgetown University Hospital Neonatal‐Perinatal Medicine Washington DC USA; ^2^ Division of Maternal Fetal Medicine Sibley Memorial Hospital Washington DC USA; ^3^ Department of Pediatrics MedStar Georgetown University Hospital Washington DC USA; ^4^ Division of Maternal Fetal Medicine Johns Hopkins School of Medicine Baltimore MD USA

**Keywords:** 19p deletion, hiatal hernia, hypotonia, intrauterine growth restriction, neonate, pyloric stenosis, small for gestational age

## Abstract

We describe the prenatal and postnatal course of an infant with a large 19p deletion. Cases such as ours will improve the knowledge of specific gene functions for every medical specialist. The goal is to allow for a more rapid diagnosis, accurate prognosis and to decrease the likelihood of complications.

## 1 INTRODUCTION

Sixteen cases with a deletion in the short arm of chromosome 19 are described in detail in the literature. Patients presented with neonatal hypotonia and developmental delay. Other findings varied significantly. We describe the perinatal course of an infant with a 3.7‐megabase deletion in the short arm of chromosome 19.

Deletions in the short arm of chromosome 19 are exceedingly rare.^1^, ^2^, ^3^, ^4^, ^5^, ^6^, ^7^, ^8^, ^9^, ^10^, ^11^While reported cases of chromosome 19p deletions have varying sizes and breakpoints and a wide range of phenotypes, all have developmental delays. We present the clinical course of a male newborn with a large deletion in the short arm of chromosome 19.

## 2 CASE REPORT

**Figure 1:**
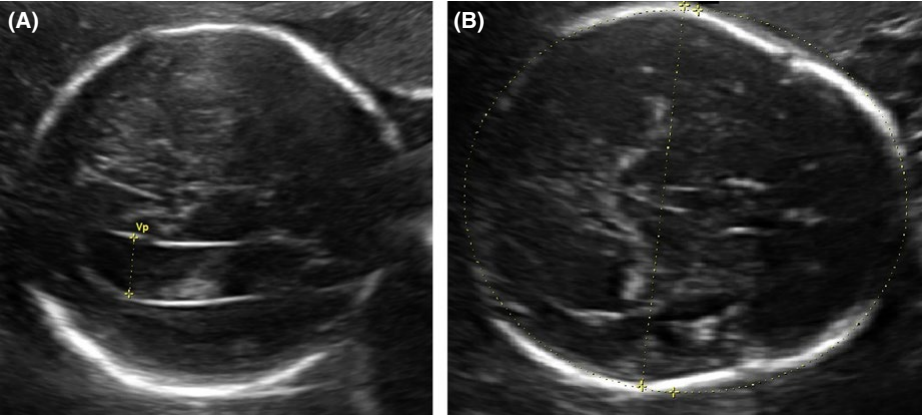
Select prenatal ultrasound images representative of (A) right lateral ventriculomegaly (12.4 mm), and (B) strawberry‐shaped head

Twin B of a spontaneous dichorionic and diamniotic twin pregnancy of a 30‐year‐old multigravida was found to have symmetrical intrauterine growth restriction (IUGR) at 25‐week gestation (estimated fetal weight (EFW) <3rd percentile, intertwin weight discordance 28%), with abnormal Dopplers indicating increased placental resistance and cranial sparing with cephalization of blood flow. Also noted were mild right lateral cerebral ventriculomegaly, small stomach, and enlarged gallbladder. Aneuploidy screening results were low‐risk, with cell‐free DNA negative for trisomy 21, 18, and 13. Amniocentesis was declined. Maternal viral serology for cytomegalovirus, toxoplasmosis, and parvovirus was negative for recent infection. From 27 weeks onward, persistently absent end‐diastolic flow of the umbilical artery was noted. Ventriculomegaly worsened to moderate range at 28 and 33 weeks, with bitemporal skull narrowing and strawberry‐shaped skull appearance noted at 31 weeks (Figure 1). Anhydramnios for Twin B was noted at 36‐week gestation. As diagnosis of aneuploidy would affect postnatal management, bladder aspiration of Twin B was performed, but cell culture was without growth. At 38 weeks, the EFW for Twin B was 1559 g, with twin discordance of 48%.

**Figure 2:**
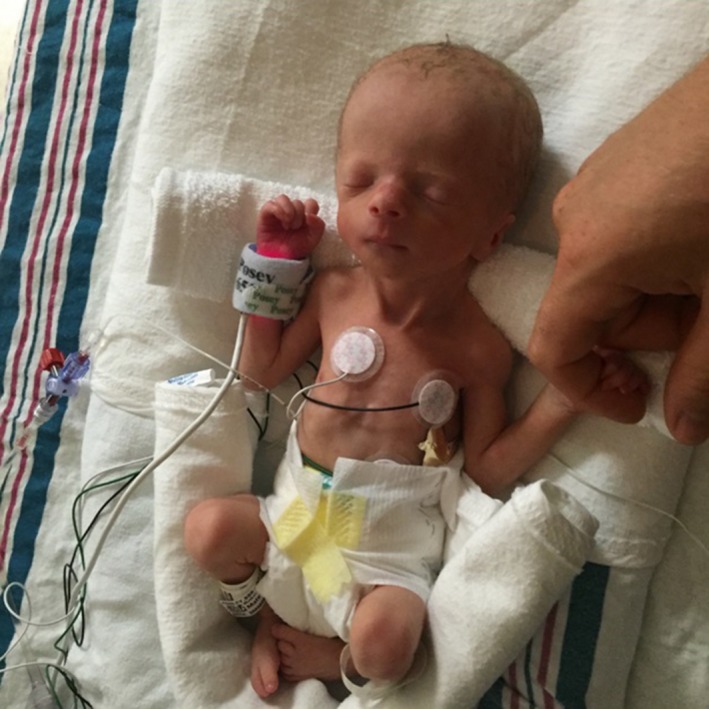
Photograph of the patient. Note the relative macrocephaly, downslanting palpebral fissures, low‐set ears, and mild micrognathia. The second finger overlaps the third finger, and the fifth overlaps the fourth finger. The nipples are wide‐spaced (left not visible)

The patient was born at 38 weeks and 3 days gestation, via scheduled repeat cesarean delivery. Apgar scores were 5, 7, and 8 at 1, 5, and 10 minutes of life, respectively. He was small for gestational age (SGA) with birthweight 1133 g (*Z*‐score: −5.49), birth length 39.4 cm (*Z*‐score: −4.43), and head circumference 29.5 cm (*Z*‐score: −3.23). Physical examination was significant for small facies with underdeveloped chin, relative macrocephaly, overlapping cranial sutures, mild crumpling of the ears, wide‐spaced nipples, second and fifth fingers of the right hand overlapping the third and fourth fingers, and a small sacral hemangioma (Figure 2). The patient developed hypoglycemia and hypophosphatemia, likely secondary to his IUGR/SGA status, which responded to modifications in intravenous fluids. He tolerated full enteral feeds by the second week of life, but due to poor oromotor skills, he was fully nasogastric tube‐dependent. Patient developed self‐resolving leukopenia. Thrombocytopenia, reaching a nadir on day of life (DOL) 2, responded to 1 platelet transfusion. Mild self‐resolving anemia was noted with a high reticulocyte count. Second anemia nadir at fourth week of life was treated with epoetin alfa, with normalization of erythrocyte indices. From DOL 5 onward, patient had a prolonged need for respiratory support, attributed to chronic microaspirations, with a waxing‐and‐waning degree of respiratory distress, which was subsequently attributed to a hiatal hernia. Gastrointestinal contrast study showed approximately half the stomach within the thoracic cavity, along with an incidental finding of inguinal hernia.

During the patient's hospital course, extensive workup was completed as part of evaluation for the chromosome 19p deletion (see Section 3). Initial head ultrasound showed a small hyperechogenic focus inferior to the third ventricle. Noncontrast brain MRI performed on DOL 23 showed normal findings. Follow‐up head ultrasound performed on DOL 50 showed resolution of the previously described echogenic focus, with a new finding of mild dilation of frontal horns of lateral ventricles. Spinal ultrasound was significant for a low lying conus and a tethered spinal cord. Whole spine MRI confirmed this finding, additionally showing 6 lumbar vertebrae. Abdominal and focused renal ultrasounds were normal. Echocardiogram was significant for suspected interrupted inferior vena cava with hemiazygos continuation to the left superior vena cava draining to the coronary sinus; no right superior vena cava was noted. Suspected cervical aortic arch was present with mild tortuosity of transverse arch without evidence of coarctation. Normal pulmonary venous drainage and a patent foramen ovale with left‐to‐right shunt were also seen. Ophthalmologic examination performed on DOL 13 showed a thin iris with rugged pupils. Mild stippling of the left retina with irregularly shaped optic disk was also noted.

On DOL 53, laparoscopy was performed to repair the hiatal hernia. Intraoperative diagnoses were: type I paraesophageal hernia with almost the entire stomach located within the chest, and a significant pyloric stenosis. The infant underwent paraesophageal hernia repair, Nissen fundoplication, G‐tube placement, and pyloromyotomy. On postoperative day four, the patient developed a firm abdomen and respiratory compromise. Abdominal X‐ray revealed intestinal pneumatosis and presence of portal vein gas. Emergent laparotomy found a 3 mm gastric perforation and necrotic small bowel. Unfortunately, the patient passed away intraoperatively. Cause of death was shock secondary to gastric perforation, peritonitis, and bowel necrosis.

## 3 METHODS AND RESULTS

Prenatal screening included First Trimester Screen (Eurofins NTD, NY) and cell‐free DNA screen (MaterniT21, Sequenom, CA). Maternal viral serologies were performed at Quest Diagnostics, VA. Prenatal fluorescent in situ hybridization (FISH) was performed by Integrated Genetics, LabCorp, NC (probes: nuc ish Xcen (DXZ1x1), Ycen (DYZ3x1), 13q14 (IRB1x1), 18cent (D18Z1x2), 21q22.13‐q22.2 (D2S342/D211S341/D21S259)x2). All prenatal imaging performed by a registered diagnostic medical sonographer with Voluson E8 (GE Healthcare, WI) and reviewed by a board certified maternal fetal medicine specialist. Neonatal FISH screen showed a normal male chromosome complement, suggesting no numerical abnormality of chromosome 13, 18, 21 or sex chromosomes. Subsequent karyotype analysis showed a normal male karyotype (46, XY). High‐resolution chromosomal microarray showed a 3.7 Mb interstitial deletion of the short arm of chromosome 19, bands p13.2 p13.12 (11 836 342‐15 505 009)x1 (Quest Diagnostics Nichols Institute, Chantilly, VA).

## 4 DISCUSSION

**Figure 3:**
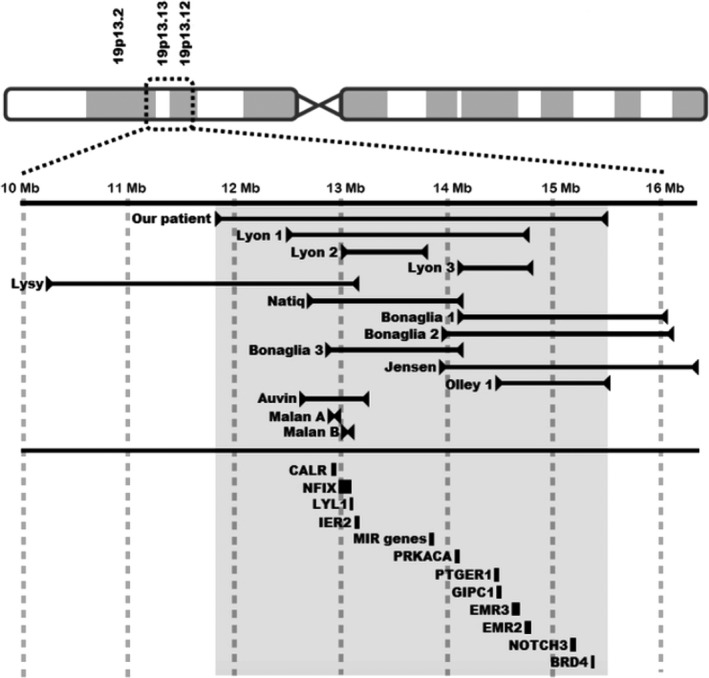
Graphic representation of sites of 19p deletion in our patient, and previously reported patients by distance in megabases (Mb) from the centromere. The shaded area corresponds to the extent of the deletion seen in our patient. OMIM‐annotated genes, pertinent to the findings seen in our patient, and their positions relative to the deletion are shown in the bottom part of the graph. The width of the bar next to the gene corresponds with the size of the gene. Hg19 positions of the genes: CALR (19:12,938,599‐12,944,489), NFIX (19:12,995,511‐13,098,795), LYL1 (19:13,099,027‐13,103,159), MIR genes (19:13,836,286‐ 13,874,807), PRKACA (19:14,091,687‐14,117,746), PTGER1 (19:14,472,465‐14,475,361), GIPC1 (19:14,477,758‐14,496,148), EMR3 (19:14,600,174‐14,674,917), EMR2 (19:14,732,696‐14,778,540), NOTCH (19:15,159,632‐15,200,980), BRD4 (19:15,236,835‐15,332,542). IER2 (GenBank accession no. NM_004907)

We present a newborn with multiple anomalies (Table 1) and a large 3.7‐Mb interstitial deletion of the short arm of chromosome 19, bands p13.2 p13.12 (11 836 342‐15 505 009)x1, which accounts for approximately 5.8% of the total length of chromosome 19 and contains at least 111 genes, including 60 OMIM‐annotated genes. There have been a total of thirteen patients with a deletion in the short arm of chromosome 19, that partially or fully overlapped with the deletion seen in our patient (Figure 3), reported in the literature with a detailed prenatal and neonatal course. Additionally, DECIPHER database provides a list of lifetime phenotypic changes for up to 41 patients with an overlapping deletion, 10 of which were classified as having “definitive or likely pathogenic deletions”.

**Table 1:** Clinical, laboratory and imaging findings seen in our patient

Prenatal findings
Severe intrauterine growth restriction with abnormal umbilical artery and middle cerebral artery Dopplers
Dilated gallbladder
Small stomach
Bitemporal narrowing/strawberry‐shaped skull
Unilateral moderate ventriculomegaly
Anhydramnios in the third trimester
Newborn physical examination
Small for gestational age
Small facies with micrognathia
Relative macrocephaly
Overlapping cranial sutures
Downslanting palpebral fissures
Low set ears
Mild crumpling of the ears bilaterally
Wide‐spaced nipples
Second and fifth fingers of the right hand overlapped the third and fourth fingers, respectively
Hypoglycemia, hypophosphatemia
Hematologic anomalies
Thrombocytopenia, transitory (25 000 per microliter)
Leukopenia, transitory (3660 per microliter)
Anemia
Neurologic anomalies
Hyperechogenic focus inferior to the third ventricle, resolved by 2 months of age
Mild dilatation of frontal horns of lateral ventricles, noted at 2 months of age
Low lying conus medullaris, tethered spinal cord
Thin iris with rugged pupils, with mild left retinal stippling and irregularly shaped optic disk
Musculoskeletal anomalies
Six lumbar vertebrae
Cardiovascular anomalies
Interrupted inferior vena cava
Hemiazygos continuation to left superior vena cava, draining into the coronary sinus
Absent right superior vena cava
Suspected cervical aortic arch
Gastrointestinal anomalies
Paraesophageal hernia, type I
Pyloric stenosis
Inguinal hernia, bilateral

The patients described in detailed case reports all had varying degree of neonatal hypotonia. In addition, 2 patients^4^, ^10^ had IUGR with head sparing, one^10^ had neonatal seizures, one^4^ had severe microcephaly, while two^4^, ^6^ presented with facial dysmorphism. Lysy et al and Lyon et al described patients with large 19p deletions and craniosynostosis. The authors raised the possibility that DAND5 and CALR genes might be involved in the pathogenesis of the neurocranial abnormality. As the same 2 genes were affected in our patient, it is unlikely that they alone would be responsible for the craniosynostosis. Additionally, the patient with a 1 Mb‐deletion in Lyon et al's report had several phenotypic features similar to our patient: antenatal onset of ventriculomegaly, postnatally noted dysmorphisms (low‐set ears and micrognathia), and bilateral optic dysplasia. The authors also raised a potential role of CACNA1A and CC2D1A genes in epilepsy and developmental delay, respectively. It is possible our patient would have developed these features, but given his early demise the full phenotype was not able to develop. Malan et al described 2 patients with a deletion of NFIX gene, who presented with drastically different phenotypes. One patient presented with Sotos‐like overgrowth syndrome, while the other presented with Marshall‐Smith syndrome (MSS). The difference was postulated to be a result of differing effects of nonsense‐mediated mRNA decay of the NFIX gene product. Many physical findings of our patient overlap with those seen in MSS: underdeveloped/small face, retrognathia, failure to thrive, respiratory distress, and pyloric stenosis. It is interesting to note that our patient's deletion affected the microRNA genes (MIR24‐2, MIR27A, MIR23A, MIR181C, MIR181D, and MIR639), potentially contributing to the MSS‐like phenotype. NFIX mutations have also been associated with pathologic thinning of the intestinal wall and diminished blood supply.^12^ Olley et al showed that haploinsufficiency of BRD4, either as part of a larger deletion or as a single gene mutation, is the likely genetic mechanism of Cornelia de Lange (CdL)‐like syndrome. Our patient had several features that would fall under either major or secondary characteristics of CdL syndrome, including: small for gestational age, microcephaly, low‐set ears, clinodactyly of the fifth finger and gastrointestinal abnormalities. It is interesting to note that of the patients described in the DECIPHER database, ones with deletions overlapping with the telomeric side of our patient's deletion had macrocephaly and optic nerve hypoplasia, while the ones overlapping with the centromeric side tended to have microcephaly and low‐set ears.

LYL1 and IER2 gene products are involved in angiogenesis, capillary formation, and vessel stabilization.^13^, ^14^ We speculate that the gastrointestinal tract in a patient with deletions of these genes would be more prone to tissue injury in a setting of peritonitis or surgery, as seen in our patient. Other potentially clinically significant genes were affected (Figure 3). CALR codes for calreticulin, which acts as a general recognition signal on apoptotic cells, thus having a crucial role in resolution of inflammation.^15^ EMR2 and EMR3 genes are involved in potentiating inflammatory responses.^16^ Deletion of these 3 genes could have led to poor healing and increased risk of gastric perforation in a setting of surgical manipulation. Steroid production is increased with increase in activity level of PRKACA‐coded protein kinase^17^ and modulated at the hypothalamic level through prostaglandin E2 receptor, coded by PTGER1.^18^ Our patient did not exhibit any hemodynamic instability that would prompt us to consider initiation of steroid replacement therapy. Nonetheless, we would recommend that with future patients who have a deletion of one or both of these genes, measurement of cortisol level be considered. It is interesting to note that despite chronic microaspiration and a remarkable number of desaturation episodes, our patient never developed any signs of pulmonary hypertension. Animal models showed that NOTCH3‐null mice did not develop pulmonary hypertension in a setting of hypoxic stimulation.^19^ It is possible that the deletion of NOTCH3 gene might have protected our patient from developing clinically significant pulmonary hypertension. GIPC1 codes for synectin, a protein crucial for cell surface receptor expression.^20^ Synectin‐null mice have abnormal pattern of arterial formation and branching, with hypoplastic dorsal aorta and stunted intersomitic vessels.^21^ Our patient's echocardiogram showed a cervical aortic arch with transverse arch tortuosity, a finding consistent with the animal models. Major vascular anomalies noted in our patient affected the large systemic veins. None of the genes deleted in our patient have previously been related to such phenotypes.

In this report, we describe the prenatal and postnatal courses of an infant with a large 19p13 deletion and address possible clinical correlation to the deletion of specific genes. Cases such as our will further improve our knowledge of specific gene functions.

## CONFLICT OF INTERESTS

Authors have no conflict of interests to declare.

## AUTHORSHIP

MC: involved in postnatal management of the patient, literature review of the 19p deletions, preclinical and clinical studies of genes involved by the deletion, writing the manuscript. JR: provided prenatal genetic counseling to the patient and coordinated prenatal aneuploidy screening and testing during the pregnancy. RSH: aided in the interpretation of the genetic anomaly, review of microarray, genes and gene location involved, and revisions of the manuscript. RD: involved in prenatal management of the mother, providing the antenatal course, and interpretation of the ultrasound findings. KK: involved in postnatal management of the patient, providing the postnatal clinical course. JT: involved in prenatal management of the mother, providing the antenatal course and interpretation of the ultrasound findings, edits, and revisions of the manuscript.
